# *Chlamydia trachomatis* and Genital Mycoplasmas: Pathogens with an Impact on Human Reproductive Health

**DOI:** 10.1155/2014/183167

**Published:** 2014-12-31

**Authors:** Sunčanica Ljubin-Sternak, Tomislav Meštrović

**Affiliations:** ^1^Teaching Institute of Public Health “Dr Andrija Štampar” and School of Medicine, University of Zagreb, Šalata 3b, 10000 Zagreb, Croatia; ^2^Clinical Microbiology and Parasitology Unit, Polyclinic “Dr Zora Profozić”, Bosutska 19, 10000 Zagreb, Croatia

## Abstract

The most prevalent, curable sexually important diseases are those caused by *Chlamydia trachomatis* (*C. trachomatis*) and genital mycoplasmas. An important characteristic of these infections is their ability to cause long-term sequels in upper genital tract, thus potentially affecting the reproductive health in both sexes. Pelvic inflammatory disease (PID), tubal factor infertility (TFI), and ectopic pregnancy (EP) are well documented complications of *C. trachomatis* infection in women. The role of genital mycoplasmas in development of PID, TFI, and EP requires further evaluation, but growing evidence supports a significant role for these in the pathogenesis of chorioamnionitis, premature membrane rupture, and preterm labor in pregnant woman. Both *C. trachomatis* and genital mycoplasmas can affect the quality of sperm and possibly influence the fertility of men. For the purpose of this paper, basic, epidemiologic, clinical, therapeutic, and public health issue of these infections were reviewed and discussed, focusing on their impact on human reproductive health.

## 1. Introduction

Sexual transmitted infections (STIs) are a major global health problem with an estimated 340 million new cases of “curable” infections occurring each year worldwide [1]. Alongside “curable” diseases that include bacterial, mycological, and protozoal infections that can be treated with appropriate chemotherapeutic agents, millions of additional cases of incurable STIs caused by viruses are also reported [2]. The most prevalent bacterial STIs are those caused by* Chlamydia trachomatis* (*C. trachomatis*). Additionally, there is growing evidence of clinical importance of infections caused by genital mycoplasmas that include various* Mycoplasma and Ureaplasma* species [3]. Although chlamydial and mycoplasmas genital infections are caused by entirely different microorganisms, there are some similarities in pathogenesis, clinical manifestations, and treatment of these infections. Their most important characteristic is the ability to cause acute complications and long-term sequelae in upper genital tract, thus affecting the reproductive health in both sexes [4–6]. The aim of this review is to acknowledge the significance of these preventable and curable infections from basic, epidemiologic, clinical, therapeutic, and public health perspective.

## 2. Bacterial Morphology and Pathogenesis

*C. trachomatis* have circular genome of 1042 kbp, which is approximately a quarter of an* E. coli* genome [7]. It also contains a cryptic plasmid 7500 bp in length [8]. Plasmid transcriptional activity can contribute to the regulation of chlamydial chromosomal gene expression [8], but direct impact of plasmid gene product on virulence is also a possibility [9]. A detection of cryptic plasmid's nucleic acid is utilized for diagnostic purposes. Mutants with a specific deletion within the plasmid that prevented* C. trachomatis* detection using a commercially available nucleic acid amplification test were described in Sweden and resulted in a concern about reliable detection methods; still, widespread problems and increase in disease severity have not been an issue [10].

*C. trachomatis* is an obligate intracellular bacterium with a unique life cycle characterized by the transformation of an extracellular, infectious elementary body (EB) in the intracellular, noninfectious, metabolically active reticulate body (RB) and vice versa. The whole cycle and its main points—such as initial ligand-receptor contact, endocytosis, and the avoidance of endocytic lysosomal pathway with the crucial role of chlamydial contact-dependent type III secretion system (TTS) in these processes—was previously reviewed by other authors [11, 12]. Furthermore, it was demonstrated that chlamydial exposure to adverse factors (e.g., penicillins or interferon gamma) induces conversion of RB into a persistent, aberrant form which does not replicate, has a reduced metabolic activity, but is still viable [13, 14]. This phenomenon is a reversible process and thus could be a possible mechanism of recurrences. Additionally, aberrant forms of RBs, with reduced major outer membrane protein (MOMP) and lipopolysaccharide (LPS) antigens, persist with high production of chlamydial heat shock protein 60 (hsp60) capable of inducing inflammation and scarring, common characteristics of chronic infection [15]. A number of chlamydial virulence factors, such as serovar-defining MOMP and TTS, define the outcome of infection and disease severity. Several types of genetic variation are found in* C. trachomatis* that impact variability and expression of virulence factors, such as high degree of variability in the exposed portions of MOMP, polymorphic TTS effectors, and amino acid substitutions in* pmp* autotransporters [16]. These strategies have been demonstrated to foster chlamydial intracellular survival, aid in the evasion of the host immune system, and form the basis for distinct chlamydial disease variations in host tissue tropism [17]. Host genetics also play a role in the disease severity. For example, women who carry specific HLA DQ and IL-10 promoter alleles that modify host immune response were found to develop TFI more frequent than control group [18].

The term “mycoplasma” is often used to refer to any members of the class Mollicutes (for the purposes of this review as well), irrespective of the fact whether they truly belong to the genus* Mycoplasma* [19]. Additionally, there are number of species in this class which are not clinically relevant, which emphasizes the need to change the generally accepted term in favor of the species. The genital tract is the main site of colonization for six species—*Ureaplasma urealyticum*,* Mycoplasma hominis*,* Mycoplasma genitalium*,* Mycoplasma penetrans*,* Mycoplasma primatum*, and* Mycoplasma spermatophilum* [19]. The latter two are considered nonpathogenic for humans. Akin to other Mollicutes, they do not possess a cell wall but instead are enclosed by a trilayered cell membrane [20]. They are smaller than conventional bacteria, both in their cellular dimensions and genome size [21]. Their genomes range from 947 kbp (711 genes) for* U. urealyticum* to 580 kbp genome (485 genes) for* M. genitalium*: the latter represents the smallest genome of a self-replicating organism presently known, demonstrating how little genetic material is actually needed to foster microbial life [22]. Several factors are important in the pathogenesis of genital mycoplasmas: (a) expression of specific adhesion proteins; (b) antigenic variation; (c) production of enzymes; and (d) facultative intracellular localization. Although the adhesions of ureaplasmas have not been characterized entirely, the evidence suggests that the receptors are sialyl residues and sulfated compounds [23]. Variable adherence-associated antigen (Vaa) and MgPa adhesion protein are believed to be major adhesion proteins in* M. hominis* and* M. genitalium*, respectively [24, 25]. Additional surface proteins, such as OppAl, are also believed to be involved in cytoadherence and may also induce ATP leakage from cells, resulting in their apoptosis. The MB (multiple-banded) antigen, a major antigen recognized during human ureaplasmal infections, and Vaa display high-frequency phase and size variation [26]. A subset of repetitive DNA elements homologous to the MgPa adhesion gene is thought to contribute to variation in the protein of the MgPa adhesion gene [27]. The variation of surface antigens is important factor in evasion of host immune response and may be related to persistence of these organisms at invasive sites. Production of enzymes such as the nuclease of* M. genitalium* or urease and immunoglobulin A1 of* U. urealyticum* provides basic compounds for their synthesis and damages the local immunity [28–30]. Facultative intracellular localization is now demonstrated for* M. fermentans*,* M. penetrans*, and* M. genitalium*, which may be responsible for protecting the organisms from antibodies and antibiotics, contributing to disease chronicity and possibly hinders* in vitro* culture [31–33].

## 3. Epidemiology

The WHO estimates that over 90 million new cases of* C. trachomatis* infections are diagnosed each year [34]. In 2012, there were 1 422 976 new cases of* C. trachomatis *infection reported in United States, revealing the rate of 456.7 per 100,000 people [35]. In the developed countries, an estimated chlamydia prevalence is highest in young heterosexual adults under 25 years of age, ranging from 3 to 6% among those who are sexually active [36, 37].

Similar to other countries,* C. trachomatis* prevalence in Croatia varies among different type of investigated population and depending on laboratory methods used for chlamydia detection. Population characteristics with a significant impact on chlamydia prevalence include age, sex, ethnicity, clinical observation (e.g., asymptomatic versus symptomatic status), and high-risk behavior (e.g., men who have sex with men—MSM). Recent study that investigated nationally representative, multistage stratified probability sample of Croatian young women and men aged 18–25 revealed 5.3% and 7.3% prevalence, respectively. Detection was performed using Roche real-time PCR assay in urine samples [38]. Earlier study that investigated* C. trachomatis* prevalence in asymptomatic men and men with symptoms of acute urethritis revealed the prevalence of 2.9% and 18.5%, respectively.* C. trachomatis* infection was diagnosed by enzyme immunoassay antigen detection method [39]. Study also revealed that the highest prevalence of 35.3% in symptomatic patients was observed among the youngest age group (18–25 years). Conflicting prevalence results of* C. trachomatis* were observed in patients with chronic prostatitis. In the period from 2003 to 2005,* C. trachomatis* was proved to be a causative pathogen in 19.3% of patients treated in one Croatian university hospital using cell culture method [40], whereas only 0.88 patients in the same hospital proved to be infected with chlamydia in the period from 2010 to 2013 when Abbott RealTime PCR test was used [41].

Akin to* C. trachomatis*, genital mycoplasmas can be transmitted through direct interaction between hosts—venereally through genitogenital or orogenital contact and vertically from mother to child (either* in utero* or at birth) [19].* Ureaplasma* spp. and* M. hominis* have been isolated from cervicovaginal specimens in 40–80% and 21–53% of women who are asymptomatic and sexually active, respectively [23]. This prevalence is somewhat lower in males [23].* M. genitalium* appears to be detected with highest prevalence in men with nongonococcal,* C. trachomatis* negative urethritis [42]. The prevalence of the organism in this group of patients ranged from 13% to 42%, and in asymptomatic men from 0% to 15% [43]. The prevalence of* M. genitalium* in symptomatic women ranged from 5 to 42% [44]. More than 20% of infants may be colonized by* Ureaplasma* spp., and infants born before term are more likely to harbor the organisms, with colonization burden declining after third month of age [23]. Less than 5% of children and 10% of nonsexually active adults are colonized with genital mycoplasmal microorganisms [19].

Research on incidence and prevalence of genital mycoplasmas in Croatia is scarce. Ružman et al. did a study on 456 pregnant women in Eastern Croatia and found positive cervical culture for* U. urealyticum*,* M. hominis*, or both in 164 (36%) of examinees [45]. One hundred and fifty-four (93.9%) of them had* U. urealyticum*, only 2 (1.2%) had* M. hominis*, and only 8 (4.9%) had both agents. In a recent study which included 1370 symptomatic and asymptomatic women of reproductive age,* Ureaplasma* spp. were identified by cultivation in 424 (34.4%) of them [46]. Subsequential genotyping of positive samples identified* U. parvum* as the predominant* Ureaplasma* species (92.6%). The prevalence of* M. genitalium* in symptomatic men in Croatia is 2.3% [47].

## 4. Impact on Female Reproductive Health

The most common clinical manifestation of* C. trachomatis* infection in women is mucopurulent cervicitis and/or urethritis [48]. Approximately half of all infected women have the infection both in cervix and the urethra, one-third in the cervix only and approximately one-quarter in the urethra only [49]. Unfortunately, a majority of infections (up to 70%) in women are asymptomatic [50], thus posing a risk for unrecognizing and subsequently not treating the infection. Left untreated, infection can lead to several complications with serious consequences for female reproductive health [51]. Spread of* C. trachomatis* from the urethra and endocervix to the upper genital tract causes pelvic inflammatory disease (PID). Herzog et al. in their mathematical model study have demonstrated that estimated fraction of chlamydia infected women that develop PID is 10% [52]. Another study conducted by Price et al. has calculated the probability of 16% that an episode of* C. trachomatis* infection will result in PID [53].

PID includes broad range of clinical syndromes: endometritis, salpingitis, tuboovarian abscess, pelvic peritonitis, periappendicitis, and perihepatitis. Diagnosis is usually based on the clinical findings, but, in severe cases of PID, laparoscopic evaluation and intra-abdominal bacterial samples are helpful for the confirmation of diagnosis and accurate microbiologic testing [54]. Acute PID can progress into a chronic form of the disease, characterized with scarification and appearance of adhesions, and further complicate with TFI and ectopic pregnancy (EP). After a single episode of PID, the relative risk for TFI is approximately 10%, and each recurrent episode of PID doubles the risk—making it almost 40% after three or more episodes [55].

Recent case-control study from two tertiary health care facilities from Benin in Nigeria demonstrated significantly higher* C. trachomatis* titers in woman with EP (48%) when compared to a control group (16.3%) [56]. In another case-control study, group of researchers from Iran looked for* C. trachomatis* in fallopian tube tissue of women with and without EP using PCR. They have detected* C. trachomatis* in 11.9% of patients with EP and none in the control group [57].

Similar to the observation that the majority of* C. trachomatis* infections in the lower female genital tract are asymptomatic, subclinical PID associated with chlamydia is also common. It represents a silent threat to female reproductive health, as it was shown that women with diagnosed subclinical PID have a 40% reduced incidence of pregnancy compared to women without subclinical PID [58]. In addition, study conducted on clinically asymptomatic women undergoing investigation of infertility and laparoscopy showed evidence of* C. trachomatis* infection in 15.9% of patients, detected by either PCR in fallopian tubes washings or EIA serology [59]. It is thought that* C. trachomatis* infection is major cause of female infertility today [60].

During pregnancy,* C. trachomatis* may cause chorioamnionitis and preterm delivery [61]. Djukić et al. demonstrated positive* C. trachomatis* antibodies and/or antigen in amniotic fluid obtained during cesarean section in 9.6% and 3.8% samples, respectively [62].* C. trachomatis* infection in pregnant women also increases the risk of low birth weight and perinatal mortality [63]. Prospective study from Belgium has shown significant association between chlamydial infection and chorioamnionitis and lower birth weight and severe neonatal infection [64]. Study from Finland clearly demonstrated that seropositivity to* C. trachomatis* in women detected during pregnancy is associated with perinatal complications [65].

The risk for vertical transmission of chlamydia is between 60% and 70% and follows the infant's passage through the birth canal, which can result in neonatal sepsis [66]. However, there is some evidence that vertical transmission can also occur* in utero*, since newborns delivered by cesarean sections have also been born infected and with intact membranes [67, 68].

*C. trachomatis* has also been found in all of the tissues of a newborn child, which suggest its invasive capacity [66, 69]. Chlamydial DNA is increasingly being detected in different tissues of neonates who died of sepsis and neonates with infection (without isolated pathogen) who died during their first week of life. In a recent study from Brazil, Hernandez-Trejo et al. demonstrated that* C. trachomatis* could play a role in the development of severe infection and in early neonatal death, similar to that observed with* M. hominis* [66]. End-point and real-time PCR of the* omp1* gene was used in this study to recognize the presence of chlamydial DNA in the paraffinized organ samples of the dead neonates. Severe neonatal infection corresponded to genotype D of* C. trachomatis*.

Finally,* C. trachomatis* infection is associated with cervical hypertrophy and induction of squamous metaplasia, thus may be a contributing cofactor in development of cervical neoplasia [70, 71]. It was shown that women with positive serum antibodies to* C. trachomatis* had a significantly increased risk for cervical cancer [72]. Study from Denmark has shown that repeated* C. trachomatis* infections increase the risk of cervical intraepithelial neoplasia (grade 3 or worse) among women with prevalent, as well as persistent high-risk HPV infection [73]. It seems that* C. trachomatis* generates an environment favorable for malignant transformation by perturbing host chromatin, DNA double-strand breaks repair, and cell-cycle regulation [74]. However not all studies could prove the association between chlamydial infection and cervical cancer development [75]. Furthermore, much of the data that support this association have been confounded by HPV coinfection. Therefore, the role of* C. trachomatis* infection in development of cervical cancer has to be further investigated and elucidated.

Genital mycoplasmas are also associated with the harmful effects on reproductive health of women and adverse pregnancy outcomes. The adverse influence of* M. hominis* on the epithelial cells of fallopian tubes in laboratory conditions was already established four decades ago [76], and this microorganism has been isolated from the endometria and fallopian tubes of about 10% of women with salpingitis [77]. In a study from Denmark on 304 infertile women, a significant correlation between TFI and seropositivity of* M. hominis* has been found and patients with TFI had a 2.13-fold higher risk of having antibodies against* M. hominis* compared with patients with normal tubes [78]. Tyagi also found that the presence of antibodies of* M. hominis* was more common in infertile women with tubal disorders [79]. Still, several newer studies did not find a positive correlation of* M. hominis* with infertility [6, 80–82].

Infertility associated with* U. urealyticum* was initially reported by Kundsin [83] and subsequently supported by other studies showing a high frequency of infection with* Ureaplasma* spp. in infertile women [84, 85]. Nonetheless, although* Ureaplasma* spp. have been isolated directly from affected fallopian tubes, it was mostly as a part of polymicrobial infection [86]. That result, along with negative serology findings and studies of inoculation of nonhuman primates and fallopian tube organ cultures, does not support a causal relationship for ureaplasmas in PID or TFI [19]. Therefore we still do not have an answer whether this particular genital mycoplasma could account for a small proportion of infertility cases or whether the relation in question is only coincidental.

The preponderance of reports implicates* U. urealyticum* and* M. hominis* in prematurity-linked conditions and* Ureaplasma* spp. are thought to be the most common organisms isolated from infected amniotic fluid and placentas [87]. In a Czech study that included 225 women with preterm premature rupture of membranes, 68% of them had cervical colonization by* U. urealyticum* compared to 17% among control patients, and 28% of them were colonized by* M. hominis* compared to 15% of controls [88].* M. hominis* was also found to be a risk factor for preterm birth after 24 weeks of gestation [89]. A study of almost 2000 women in Belgium found a preterm birth rate of 4.9%, and 53.6% of those with premature delivery showed colonization with* Ureaplasma* spp. [90].

Another study of 150 women with premature rupture of membranes reported that* U. urealyticum* was present in 96% of subjects, compared to only 32% of women who did not experience membrane rupture [91]. A study of placental cultures from Japan found that among 151 placentas from pregnancies that ended with spontaneous preterm birth before 32 weeks of gestation, 63 were culture positive for* Ureaplasma* spp. and 83% of these showed histologic chorioamnionitis, whereas only 30% of culture negative placentas showed signs of chorioamnionitis [92]. Authors from Austria indicated that there is a dose-dependent inflammatory response inside the amniotic cavity to* U. parvum* and that this is related not only to premature membrane rupture, preterm labor, and histologic chorioamnionitis, but also to bronchopulmonary dysplasia and early onset sepsis in the baby [93]. Kataoka et al. indicated that a detection of* U. parvum* in a vagina was associated with late abortion and early preterm birth [94].

Since Taylor-Robinson et al. demonstrated in 1987 that primates inoculated with* M. genitalium* develop both salpingitis and lower genital tract pathology [95], a myriad of other studies supported the theory that this bacterium has a role as an etiologic factor in PID. Bjartling et al. did a nested case-control study in Sweden among women undergoing the termination of pregnancy [96]. Of the 49 women with PID, 12.2% were positive for* M. genitalium* (compared with a 2.4% positivity in the control group); hence the organism in this study was strongly associated with posttermination PID, and a causal relationship was suggested. In a different study from the same authors on a heterogeneous population of women attending a gynecological outpatient service,* M. genitalium* was a strong and independent risk factor for both PID and cervicitis [97].

Positive association of* M. genitalium* with short-term PID treatment failure was also described by Haggerty et al. on samples from the PID Evaluation and Clinical Health (PEACH) Study [98, 99]. In contrast, one prospective study following female sex workers in Kenya over a period of 36 months failed to find an association of* M. genitalium* infection with PID [100]. Still, taking into account the persistent nature of* M. genitalium* (comparable to other STDs), it is possible that the duration of the follow-up period and high percentage of loss to follow-up were inadequate to detect incident PID. It has to be noted that the clinical diagnosis of PID includes several variable signs that frequently do not correlate with laparoscopic findings, which certainly contributes to inconsistency among PID studies and could influence the associations with* M. genitalium* infection.

No association between* M. genitalium* and EP was established thus far [101], and its role in adverse pregnancy outcomes is still unclear [102]. Several studies have shown an independent association of* M. genitalium* with preterm birth [103–105], although no other syndromes have been linked to the infection with this organism [106, 107]. In a group of 915 women from the United Kingdom, Oakeshott et al. demonstrated an association between* M. genitalium* and preterm birth [103]. Edwards et al. found that* M. genitalium* was independently associated with spontaneous preterm delivery on a cohort of 137 women [104].

Research on* M. genitalium* as a cause of female infertility has shown a high correlation. Two studies from Denmark have found a significant association between women with specific serum antibodies to this bacterium and laparoscopically established tubal infertility. Clausen et al. examined sera for antibodies to* M. genitalium* by immunoblotting from 308 women presenting to an infertility clinic in Denmark using laparoscopically confirmed tubal occlusion as the diagnostic criterion [108]. The results revealed that the relative risk of tubal factor infertility in women with* M. genitalium* was 3.8. A strong antibody response against* M. genitalium* without the signs of current or chronic infection was found in women with TFI in the study of Svenstrup et al., indicating that preceding infections with this microorganism may have caused permanent injury and occlusion of the fallopian tubes [109].

In a study by Grześko et al. of 74 Polish women attending an infertility clinic,* M. genitalium* was detected by PCR more frequently in cervical swabs from infertile patients when compared to healthy, fertile women [110], indicating that endocervical swabs can forecast upper tract infection. Baczynska et al. have proven that the presence of* M. genitalium* in the human fallopian tubes organ culture affected the epithelium and resulted in cilia destruction, although the damage was not so extensive when compared to the damage caused by* C. trachomatis* [111].

## 5. Impact on Male Reproductive Health

Approximately half of the men infected with* C. trachomatis* show no symptoms of infection [112]. Nongonococcal urethritis is the most common clinical presentation of* C. trachomatis* infection in males, which can be complicated with epididymitis and orchitis—especially in young men [39, 113–116]. Although chlamydia is well recognized and accepted cause of male urethritis, epididymitis, and orchitis, the role of* C. trachomatis* in pathogenesis of prostatitis is controversial. There are studies that suggest that* C. trachomatis* is the causative agent in one-fifth to one-third of patients with prostatitis [40, 117–119]. In our opinion, chlamydial prostatitis should be diagnosed carefully, considering the symptoms, clinical findings, obtained clinical sample (e.g., semen, urine, expressed prostatic secretion, and prostatic tissue), and employed laboratory methods. Our recent study demonstrated that the significance of* C. trachomatis* in etiology of prostatitis has been overemphasized, mostly as result of using nonspecific methods for laboratory diagnosis of* C. trachomatis* infection [41]. In addition, chronic prostatitis caused by chlamydia could be very tricky for laboratory diagnosis and treatment, due to the evidence of persistent forms that have been reported after treatment of chronic prostatitis with antimicrobial drugs [120].

Ascending chlamydial infection can potentially result in scarification and occlusion of the canalicular system of male genital tract and thus influence male fertility, but it seems to be a very rare phenomenon [49, 121]. More important is the possible influence of* C. trachomatis* infection on sperm quality. A number of studies investigated the relationship between* C. trachomatis* infection and semen quality with contradictory results [122–125]. Nevertheless, some of them suggest that exposure to* C. trachomatis* can affect sperm function and induce premature sperm death [126, 127].

It is estimated that 15% of male infertility is related to genital tract infection [128]. Among the causative factors,* U. urealyticum* is one of the most frequently encountered species [129]. Since 1967, the ureaplasmas have been linked to the etiology of male infertility [130], especially after Friberg and Gnarpe [131] demonstrated for the first time a higher frequency of ureaplasmas in the semen of men with unexplained infertility (76%) when compared with fertile men (19%). The presence of* U. urealyticum* could cause dysfunction of accessory sex glands, and the abnormality of their secretion can lead to a change of seminal characteristics [132]. Xu et al. reported that* U. urealyticum* infection reduced spermatozoa motility and increased their abnormality rate [133]. A study from Poland found that deteriorated semen density, sperm vitality, and progressive motility of spermatozoids were associated with* U. urealyticum* [134]. This infection was also associated with higher semen viscosity and lower semen pH value, and sperm concentration was lower in positive subjects in the study of Wang and coauthors [129].

Recent studies also point to the detrimental effects of* U. urealyticum* on the conventional sperm parameters. The presence of this microorganism was related to lower mean sperm concentration and lower vitality of spermatozoa in the study of Liu et al. which included a total of 621 infertile and 615 fertile men [125]. Progressive motility and vitality were significantly lower in men positive to this microorganism than in men without it in a study from the Republic of Korea conducted in a fertility clinic [135]. Statistically significant decrease in the integrity of sperm plasma membrane in patients with* U. urealyticum* has also been recently demonstrated by Chinese researchers [136]. In addition, recent study from Montenegro showed that treating the infection resulted in the increase of the sperm concentration itself with the significant improvement of progressive motility, although being without the effect on the viability of the spermatozoa [137].

Several of the abovementioned studies researched the influence of* M. hominis* on semen parameters as well. In a study from Lee et al. low total motility and total motile sperm count were significantly related to the presence of this mycoplasma [135]. In a study among 250 unselected infertile men, there were a significantly higher percentage of patients with oligoasthenoteratozoospermia or asthenozoospermia alone in the group infected with* M. hominis* compared to noninfected, infertile patients [138]. The presence of* M. hominis *DNA in semen samples was associated with low sperm concentration and abnormal sperm morphology in a study from Tunisia, although the mean values of pH, total volume, vitality, motility, and polymorphonuclear count were not significantly related to the detection of genital mycoplasmas [139]. Sequential sectioning of spermatozoa infected with* M. hominis* revealed the intracellular location of this bacterium within cytosolic spaces of head and midpiece regions, suggesting that this kind of interaction could lead to subtle damage that can have implications for long-term male or couple's infertility [140].

More research on* M. genitalium* regarding the effect on semen parameters and male infertility is needed. Thus far this microorganism has been isolated from semen specimens and its ability to attach to human spermatozoa has been shown by X-ray microscopy [141, 142]. In a study from Gdoura et al., the concentration of spermatozoa among the male partners of infertile couples with M. genitalium DNA in semen specimens was significantly lower when compared to male partners without this microorganism [139]. On the other hand, in a study on 127 infertile and 188 fertile men in Kuwait, Al-Sweih et al. concluded that no significant association between M. genitalium and diminished fertility exists, although they did note that genital mycoplasmas appeared to negatively influence quality of the semen [143].

## 6. Laboratory Diagnosis

Since both chlamydial and mycoplasma infections may not show specific symptoms and are often indistinguishable or asymptomatic, laboratory diagnosis is necessary in order to establish the correct etiology.

### 6.1. Culture Methods

**Figure 1:**
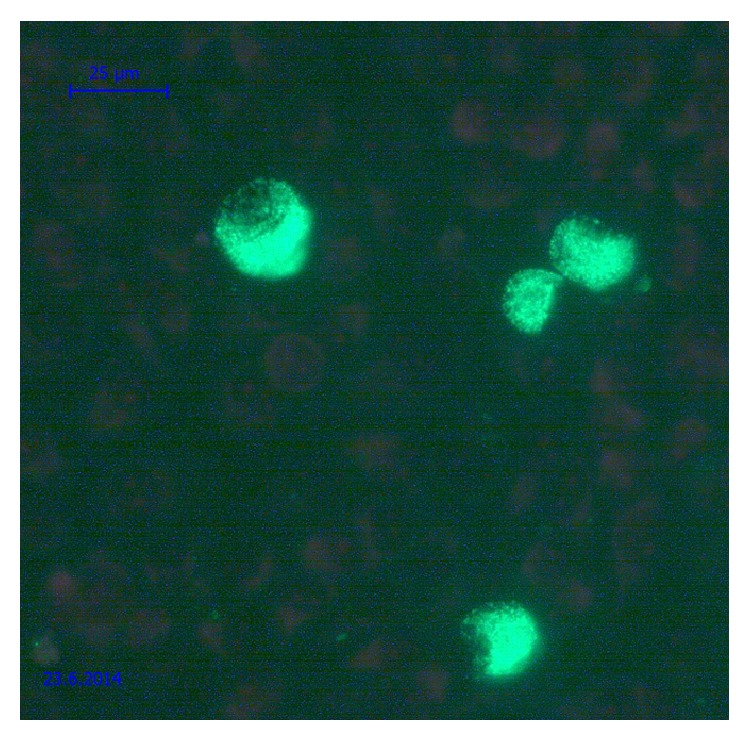
*Chlamydia trachomatis* inclusions in McCoy cell culture detected by fluorescein labeled monoclonal antibodies against lipopolysaccharide antigen. Note the lens-like uncolored region on the one side of each inclusion that presents displaced nucleus of infected cell.

As chlamydia is an obligate intracellular pathogen, it requires living cells for its multiplication. Isolation in cell culture traditionally has been considered as a “gold standard” for many years, but with the advent of molecular methods its role has been challenged. Such culture method is technically demanding, labor-intensive, cumbersome, expensive, and—most importantly—less sensitive when compared to the nucleic acid amplification tests (NAATs) [144]. Specificity of the culture method approaches 100% when fluorescein-labeled monoclonal antibodies are used for the detection of inclusions (Figure 1). Sensitivity in experienced laboratory approaches 85% compared with NAATs [145]. Another disadvantage of this method is that it requires perfectly organized cold chain of transportation in order to deliver viable microorganism to the laboratory [146]. However, because an isolation of living microorganism is the definitive method for the diagnosis, culture remains the method of choice in terms of medicolegal investigations and follow-up after completed therapy [145]. Additionally, it also serves for determining the antimicrobial sensitivity of* C. trachomatis* [147, 148].

As with chlamydia, culture is also regarded as a “gold standard” for the detection of recoverable organisms like* M. hominis* and* Ureaplasma* spp.; nevertheless, a low sensitivity when compared to polymerase chain reaction (PCR) assays has been reported repeatedly [149, 150]. Culture is labour-intensive and time consuming, as it entails the use of an enrichment broth for up to seven days, followed by subculturing onto solid media [150, 151]. The development of commercially available diagnostic assays, which are based on liquid broth cultures, provides faster and more user friendly alternatives to conventional culture methods for the detection of genital mycoplasmas [47, 151]. Enriched liquid broth that contains urea, arginine, and phenol red indicator is observed for eventual changes of the colour and allows subculture to solid media with subsequent recovery and identification of both* M. hominis* and* Ureaplasma* spp. [47]. The culture of* M. genitalium* is even more difficult and time consuming, and no liquid broth culture methods exist, which is the reason why cultivation of this organism is employed for research purposes only [152]. All of that hinders the possibilities for microbial susceptibility testing of genital mycoplasmas.

### 6.2. Antigen Detection Methods

There are several commercially available antigen detection methods for diagnosis of chlamydial infection with two main approaches: direct fluorescein test (DFA) and enzyme linked immunosorbent assay (ELISA). Those tests do not require stringent conditions for specimen transportation. The DFA test is the only diagnostic test that permits simultaneous assessment of specimen adequacy by visualization of epithelial cells present in the smear [146]. In relation to culture, the sensitivity and specificity of DFA tests that use antibodies to MOMP are 80–90% and 98-99%, respectively [153]. Most of the ELISA tests detect chlamydial LPS which is more soluble than MOMP, although it can cross-react with other gram-negative bacteria [154]. Their sensitivity and specificity range from 62 to 96% and from 86 to 99%, respectively, in comparison to cell culture [155]. Antigen detection techniques have not been developed for genital mycoplasmas [19].

### 6.3. Molecular Methods

Development of the NAATs has been the most important advancement in the field of chlamydial diagnosis due to their high sensitivity; potentially, they are capable of detecting as little as a single gene copy [156]. Such characteristic enabled the usage of noninvasive specimens like urine. Transportation is also not a crucial issue, as molecular testing does not require living organism. All these characteristics were an important enhancement, especially for screening purposes. Currently, three NAATs dominate molecular diagnostics of* C. trachomatis*: Roche Cobas TaqMan CT assay that targets both the cryptic plasmid and the* omp1* gene, the Abbott RealTime CT m2000 PCR that targets two parts of the cryptic plasmid, and the Gen-Probe Aptima Combo 2 that targets the 23S rRNA molecule [157]. Comparing to other NAATs, the Roche TaqMan assay shows superior specificity (100%), but with sensitivity estimated at 82.4%. All of the mentioned assays can successfully detect the new variant strain of* C. trachomatis*, described in Sweden in 2006 [10].

PCR is the most broadly applied NAAT for detection of genital mycoplasmas as well and has been adapted to identify antimicrobial resistance determinants or to evaluate genetic relatedness of clinical isolates [158]. Modern laboratories today have eliminated conventional PCR in favour of real-time PCR, using the Roche LightCycler for detection and identification of all the major human mycoplasma species due to its advantages in accuracy, quantitation, and turnaround time [159]. The enhanced specificity of real-time PCR (compared with conventional PCR) is chiefly because of the use of a third oligonucleotide probe that binds to the target sequence, thus minimizing the potential cross-reactions [160]. Publications describing real-time PCR for detection and characterization of* M. hominis* and* Ureaplasma* spp. have used previously mentioned Roche LightCycler 2.0, as well as Applied Biosystems Prism 7900HT and the Bio-Rad iCycler iQ [159]. For slow-growing species, such as* M. genitalium*, molecular-based detection is the only practical way for rapid diagnosis, although commercial assays available for detection (AmpliSens Mycoplasma genitalium-EPh PCR kit and Euroclone Duplic*α* RealTime) are still pending FDA approval and are still mostly used for research purposes [47].

Molecular technology also enables simultaneous detection of more than one microorganism. Multiplex real-time PCR was found to be an equivalent or superior modality for the diagnosis of STIs [161]. In a recent study by Korean authors, Anyplex II multiplex real-time PCR for seven different pathogens had 100% sensitivity and high specificity for the detection of* C. trachomatis*,* M. genitalium*, and* M. hominis*, and it was also useful for discriminating between* U. urealyticum* and* U. parvum* [162]. Simultaneous direct identification of* C. trachomatis*, genital mycoplasmas, and several other genital microorganisms in voided urine using multiplex PCR-based reverse line blot assays had also been recently described [163]. Sexually transmitted infection profiling (STIP) assay that detects 18 sexually transmitted infections (among them* C. trachomatis*,* M. genitalium*,* M. hominis*,* M. spermatophilum*,* U. urealyitcum*, and* U. parvum*) using a multiplex PCR followed by Luminex bead-based hybridisation has been described with an overall concordance of 95–100% with commercially available quantitative PCR tests [164].

### 6.4. Serology

Generally, serological tests in the diagnosis of genital tract infections caused by* C. trachomatis* are not useful, as the antibodies elicited by* C. trachomatis* are long lived and positive antibody test usually cannot distinguish previous from current infection. However, serology may have some diagnostic significance in investigations of woman with PID, TFI, and spontaneous miscarriage [165], and negative result may have predictive value in infertile women [166].

Higher anti-cHSP60 antibody responses in women with tubal occlusion and TFI caused by* C. trachomatis* have been demonstrated in contrast to women without tubal pathology [167, 168]. These findings have led to the development of a commercial ELISA screening test based on cHSP60 (Medac, Hamburg, Germany) [169]. Still, studies evaluating the diagnostic potential of the Medac cHSP60 ELISA test have revealed conflicting results, thus the ability of this assay to distinguish various* C. trachomatis* disease stages may be limited [169, 170]. Australian study by Collet et al. demonstrated that the use of four chlamydial antigens (CT157, CT423, CT727, and CT396) could potentially facilitate earlier diagnoses in women suffering from tubal occlusion and other pathologies of the upper genital tract [171]. They were found to be capable of discriminating between the infection and disease sequelae, such as tubal infertility. Sensitivity of 80% and specificity of 86% have been noted for this assay.

Serological test methods for* Ureaplasma* spp.,* M. hominis*, and* M. genitalium* include enzyme immunoassay, microimmunofluorescence, and metabolism inhibition, but the ubiquity of these microorganisms in healthy people makes the interpretation of antibody titers against them challenging [19]. As the most serious outcome of tubal scarring caused by* M. genitalium* can be long-term infertility, serological studies represent the best choice for addressing the issue whether this organism is a cause of TFI and can also be useful in determining recent or long-term infections (i.e., by comparing IgM and IgG antibodies) [102]. The cross-reactions between* M. genitalium* and* M. pneumoniae* [172] have hampered the use of serology for diagnosis and epidemiological studies, but Wang et al. developed and evaluated a Triton X-114 extracted lipid-associated membrane protein (LAMP) assay without evident cross-reactivity [173]. Thus the LAMP-EIA method adapted by using two different strains of* M. genitalium* as antigen (in order to represent different antigenic variants of the pathogen) is currently used in most serological evaluations of tubal disorders and PID caused by this microorganism [101].

## 7. Treatment and Antimicrobial Resistance

Due to the absence of peptidoglycan layer in the cell wall of both* Chlamydia* and genital mycoplasmas, antimicrobial drugs that interfere with protein or nucleic acid synthesis (e.g., tetracyclines, macrolides, and quinolones) are recommended for the treatment. However, there are some specificities and general trends regarding susceptibility of each species (Table 1).

To date,* C. trachomatis* resistance has not been of great concern as majority of studies report excellent sensitivity of chlamydia* in vitro* and* in vivo*—even in countries with high antibiotic consumption rate [147, 174, 175]. However, clinical treatment failures have been reported and some of them linked to multidrug-resistant* C. trachomatis* strains which all demonstrate heterotypic resistance, which is a form of phenotypic resistance where a small proportion of an infecting microbial species is capable of expressing resistance [176–178]. Some researchers associate this phenomenon with chlamydial aberrancy, concluding that such phenotypic antibiotic resistance may be a possible adaptive behaviour of* C. trachomatis* under antibiotic stress rather than stable genetic resistance mechanism [179].

Although macrolides are considered as drugs of choice for treating mycoplasmal infections, caution is necessary since* M. hominis* shows intrinsic resistance to the C14 and C15 macrolides (erythromycin and azithromycin) [180]. On the other hand,* Ureaplasma* spp. is naturally resistant to lincosamides (e.g., clindamycin) [181]. Acquired resistance to macrolides of these species is associated with mutations in the 23S rRNA gene [182, 183], whilst resistance to tetracyclines is related to the presence of the mobile* tet(M)* genetic element [184, 185]. Fluoroquinolones (particularly fourth-generation moxifloxacin) remain very effective against genital mycoplasmas, but resistance patterns show an increasing tendency and they are restricted to nonpregnant patients [186].

The rate of bacteriologic failure after treatment of* M. genitalium* with doxycycline is high and could lead to the development of chronic illness; hence this antibiotic is not recommended [187, 188]. Both the single dose of 1 g and the extended treatment with 1.5 g azithromycin are efficient and they do not significantly differ, but, due to the increased selection of resistant clones, extended treatment with this drug is recommended [189]. However, as macrolide resistance is on a steep rise [190] and ciprofloxacin and levofloxacin do not show adequate efficiency [191], moxifloxacin is currently recommended as a drug of choice in cases of azithromycin-resistant* M. genitalium* [188, 191]. However, authors from Australia and Japan reported cases of clinical and microbiological moxifloxacin treatment failure in infections caused by this microorganism [192, 193]. In the foresight, sitafloxacin could be a promising agent for* M. genitalium* infections [194].

## 8. Public Health Dimension and Conclusion

Chlamydial and genital mycoplasma infections are the most important preventable causes of female infertility and adverse pregnancy outcomes. When they ascend, both of the infections can result in PID—a leading cause of TFI and EP. Screening could improve outcomes of infections by identifying and treating them before progressing to PID (direct effect) or by reducing their transmission (indirect effect) [195]. An improved understanding of the natural history of* C. trachomatis* and genital mycoplasma infections is essential to boost the control efforts [51, 196]. Natural history studies would ideally help to better elucidate the incidence and timing of PID and tubal damage, resulting in long-term sequelae after untreated infections [197]. Such assessments would have to be done in diverse populations, including women with asymptomatic prevalent infection without the indication for testing, apart from screening. Also, further research is required to understand the dynamics of* C. trachomatis* and mycoplasma coinfections.

A critical component of research addressing natural history and the impact of chlamydia and mycoplasma screening is our aptitude to accurately measure the sequelae of these infections. We desperately need better, noninvasive tools for measuring the impact on human reproductive health. Diagnosis of acute PID is extremely subjective, insensitive, and nonspecific [198]. Infertility has multiple possible causes and may not be recognized for years after a chlamydial or mycoplasmal infection has resulted in a tubal damage, as the affected woman may not have tried to become pregnant. Thus it is essential to have tools to more accurately assess the sequelae observed as end-products (namely, PID, EP, and infertility) and also to noninvasively detect the prevailing pathophysiologic processes that forecast those sequelae [199]. The issue of morbidity and possible influence of those microorganisms on male fertility is still not completely clear; hence more research is needed in that direction to establish effective prevention programs.

Vaccination could be considerably more effective than other biomedical interventions in controlling epidemics of* C. trachomatis* and genital mycoplasma infections. Administrating a protective vaccine to adolescents before their first sexual experience could induce a significant reduction in prevalence, which could not be achieved by screening teenagers (even with the full coverage) [200]. Unfortunately, no fully or partially protective vaccines are available despite prior attempts to develop one in case of* C. trachomatis* [201]. The immunological features of the genital system and the tropism of* Chlamydia* for mucosal epithelial cells underline the necessity of inducing both mucosal and systemic protective responses in an ideal vaccine [202]. The difficulty also arises because the male reproductive tract is an immune-privileged site that can be disrupted, possibly affecting spermatogenesis if ill-suited inflammatory responses are provoked [4]. Therefore for a better understanding of the immunologic, host, and organism factors that have a role in pathogenesis and the development of sequelae, a pursuit for relevant clinical markers and a viable vaccine could ultimately help guide targeted screening and control efforts of these important pathogens.

## Figures and Tables

**Table 1:** General trends in *C. trachomatis* and genital mycoplasmas drug susceptibility.

	*C. trahomatis *	*M. hominis *	*U. urealyticum *	*M. genitalium *
Doxycycline	+	+	+	−
Azithromycin	+	−	+	+
Erythromycin	+	−	+	−
Clindamycin	−	+	−	−
Ciprofloxacin	+	+	+	−
Levofloxacin	+	+	+	−
Moxifloxacin	+	+	+	+
